# Ecological Niche and Geographic Distribution of Human Monkeypox in Africa

**DOI:** 10.1371/journal.pone.0000176

**Published:** 2007-01-31

**Authors:** Rebecca S. Levine, A.Townsend Peterson, Krista L. Yorita, Darin Carroll, Inger K. Damon, Mary G. Reynolds

**Affiliations:** 1 Centers for Disease Control and Prevention, Poxvirus Program, Atlanta, Georgia, United States of America; 2 Natural History Museum and Biodiversity Research Center, University of Kansas, Lawrence, Kansas, United States of America; 3 Centers for Disease Control and Prevention, Division of Viral and Rickettsial Diseases, Atlanta, Georgia, United States of America; North Carolina State University, United States of America

## Abstract

Monkeypox virus, a zoonotic member of the genus *Orthopoxviridae*, can cause a severe, smallpox-like illness in humans. Monkeypox virus is thought to be endemic to forested areas of western and Central Africa. Considerably more is known about human monkeypox disease occurrence than about natural sylvatic cycles of this virus in non-human animal hosts. We use human monkeypox case data from Africa for 1970–2003 in an ecological niche modeling framework to construct predictive models of the ecological requirements and geographic distribution of monkeypox virus across West and Central Africa. Tests of internal predictive ability using different subsets of input data show the model to be highly robust and suggest that the distinct phylogenetic lineages of monkeypox in West Africa and Central Africa occupy similar ecological niches. High mean annual precipitation and low elevations were shown to be highly correlated with human monkeypox disease occurrence. The synthetic picture of the potential geographic distribution of human monkeypox in Africa resulting from this study should support ongoing epidemiologic and ecological studies, as well as help to guide public health intervention strategies to areas at highest risk for human monkeypox.

## Introduction

Monkeypox virus, a member of the genus *Orthopoxviridae*, can cause a serious, smallpox-like illness in humans. Since the global eradication of smallpox in 1977, monkeypox virus has been considered the most problematic orthopoxvirus as regards human health [1]. Nonetheless, little is known about the geographic distribution, ecology, natural reservoir, or intermediate zoonotic host(s) of the virus. Human monkeypox is endemic to forested areas of West and Central Africa, and is thought to be transmitted to humans through contact with infected animals, and through person-to-person spread [1]–[3].

Considerably more is known about human monkeypox disease occurrence than about natural infections in non-human animal hosts. More than 30 isolates have been cultured from human clinical specimens, but monkeypox virus has been isolated only once from a wild animal–a moribund rope squirrel (*Funisciurus anerythrus*) captured in the Democratic Republic of the Congo (DRC) [4].

A further question is whether West and Central African monkeypox viruses occupy the same ecological niche. The zones of West and Central African endemicity are geographically discontinuous, and while hundreds of human cases have been reported in Central African countries only a handful have been identified in West Africa [5]–[13]. Phylogenetic analyses suggest that West and Central African monkeypox virus strains form distinct clades, each with variant subtypes [14], [15] and several significant biological features have been shown to differ between viruses in the two clades [e.g., Central African strains are more likely to be transmitted among humans, and to cause more severe illness, than West African strains [15]. Whether these apparent disparities reflect biological divergence sufficient to translate into distinct ecological regimes (or animal reservoirs) bears investigation.

This study aims to develop predictive models describing the ecological requirements and potential geographic distribution of human monkeypox in Africa using an ecological niche modeling approach [16]. This approach provides a foundation for testable hypotheses regarding the geographic range of human monkeypox, and provides a useful guide for identification of potential reservoir hosts associated with transmission to humans. Results of this study will have implications for understanding the ecology, natural history, and epidemiology of this virus.

## Materials and Methods

### Human Monkeypox Occurrence Data

Locations of known occurrences of human monkeypox in the endemic regions of Africa were compiled though comprehensive literature search and analysis of Centers for Disease Control and Prevention (CDC) and World Health Organization (WHO) data collections compiled from outbreak investigations and surveillance activities. For this study, a human monkeypox case was defined as a published report or a non-redundant, unpublished case (with supporting laboratory evidence) recorded in CDC or WHO monkeypox data collections. Cases recorded by WHO during the years 1970–1986 were classified based on results of findings from electron microscopy, virus culture, and/or serology (P. Formenty, personal communication 2004). CDC's current case definition requires laboratory evidence of monkeypox virus in clinical specimens demonstrated via polymerase chain reaction (PCR), electron microscopy, or tissue culture [17].

For ecological niche modeling (ENM) analyses, occurrence locations were considered only once, with no weighting to account for multiple cases at single locations, thus minimizing potential confounding attributable to (rare) instances of person-to-person transmission. Our tally of spatially unique human monkeypox cases was 371 distinct locations. However, given geographic complexities and incomplete gazetteer databases, not all named locations (villages) could be assigned exact point coordinates— only 156 human monkeypox occurrences could be assigned geographic latitude and longitude coordinates accurate to at least one minute.

### Geographic Data

Environmental data sets input into Genetic Algorithm for Rule-Set Prediction (GARP) for ENM came from three principal sources. (1) Climatic data averaged over the period 1961–1990, including data layers (‘coverages’) summarizing mean annual temperature, mean maximum monthly temperature, mean minimum monthly temperature, diurnal temperature range, mean annual precipitation, wet days, and ground frost days, were drawn from the Intergovernmental Panel on Climate Change (IPCC; native resolution ∼50×∼50 km; http://ipcc-ddc.cru.uea.ac.uk/). (2) Land-surface data summarizing elevation, aspect, water flow accumulation, water flow direction, and compound topographic index (a measure of the tendency of water to pool) were obtained from the U.S. Geological Survey's Hydro-1K data set (native resolution 1×1 km; http://edc.usgs.gov/products/elevation/gtopo30/hydro/). Finally, (3) summarized land cover across Africa were drawn from the University of Maryland Global Land Cover Facility (http://glcf.umiacs.umd.edu/index.shtml). All data layers were generalized to a pixel resolution of ∼10×∼10 km (0.1×0.1°) for analysis, in view of some georeferencing imprecision for occurrence localities.

### Ecological Niche Modeling

We define the ecological niche of a species as the set of environmental conditions within which it is able to maintain populations without immigration [18], [19]. Ecological niches and associated potential geographic distributions can be approximated via correlative approaches that relate known point-occurrence data to digital GIS data layers summarizing spatial variation in relevant environmental dimensions [20]. The algorithm used for generating ENMs was the GARP (GARP version 1.1.3) [21], [22]. GARP is an evolutionary-computing method that builds models based on non-random associations between known occurrence points for species and sets of GIS coverages describing the ecological landscape. Occurrence data are used by GARP as follows: 50% of occurrence data points are set aside for an independent test of model quality (extrinsic testing data); 25% are used for developing models (training data); and 25% are used for tests of model quality internal to GARP (intrinsic testing data). Distributional data are converted to raster layers, and by random sampling from areas of known presence (training and intrinsic test data) and areas of ‘pseudoabsence’ (areas lacking known presences), two data sets are created, each of 1250 points; these data sets are used for rule generation and model testing, respectively [21], [22].

The genetic algorithm produces a logic model, rather than a strictly derived mathematical model. An initial condition (first rule applied) is created in GARP by application of a single inferential tool randomly selected from a defined set. This set includes 4 basic rule types (bioclimatic rules, atomic rules, range rules and logistic regression), each of which implements a different method for building prediction models. Subsequent combinations of rules with specially defined operators (e.g. crossover, mutation) are then used to modify the initial rules, and through iteration and optimization, models are “evolved”. After each modification, the quality of the rule is tested (to maximize both significance and predictive accuracy) and a size-limited set of the best rules is retained. Because rules are tested based on independent data (intrinsic test data), performance values reflect the expected (general) performance of the rule, an independent verification that gives a more reliable estimate of true rule performance. The final result is a set of rules that can be projected onto a map to produce a potential geographic distribution for the species under investigation.

To produce a final prediction model (map), 10 individual GARP models were created, each with 100,000 maximum iterations and a convergence criterion of 0.0001. The final prediction maps were produced by summing these 10 high-quality models. Color gradations are used to indicate the proportion of times out of 10 that specific areas (pixels) were included in the predicted distribution of human monkeypox.

Model quality was evaluated via the independent testing data subsets that were set aside prior to modeling. In general we compared observed coincidence between model predictions and test data with random expectations. A *X* ^2^ test was used to compare observed success in predicting distributions of test points with those expected under random (null) models (proportional area predicted present provides an estimate of occurrence points correctly predicted were the prediction to be random with respect to the distribution of the test points).

### Identification of Key Environmental Factors

To assess the relative importance of the individual ecological parameters, a jackknife procedure was performed, involving construction of a series of ENMs, each systematically omitting one of the *n* layers, following procedures outlined by Peterson and Cohoon [23]. This manipulation resulted in *n* - 1 maps, each representing the predicted distribution of the disease without consideration of the information in a particular parameter; effects of these manipulations were summarized by a calculation of percent difference (across all pixels in the map) from the map produced using all variables.

The empirical contribution of the information contained in each layer toward creation of the comprehensive ENM (i.e., the statistical significance of each parameter within the overall model) was assumed using a single sample Student's *t*-test (H_0_ = 0) to evaluate differences in the mean number of pixel matches between the comprehensive ENM (based on *n* variables) and each derived ENM (based on *n*-1 variables). To accomplish this test, each pixel in the map (N = 654,754) was assigned a value between 0 and 10 corresponding to the frequency of positive prediction in the 10 summed models (see above). The mean difference in predicted level for matched pixels across the population of pixels in the comprehensive versus derived ENMs was then compared to a hypothesized value of zero (signifying that the derived and comprehensive ENMs were identical). Kappa statistics were also used to assess levels of agreement between the comprehensive and derived ENMs.

### Model Robustness

To provide a test of the ability of our ENMs to predict the distribution of human monkeypox cases into areas from which no input data are present, we used a spatially stratified subsetting procedure that we term the ‘quadrant test’ (see [24] for another example) to challenge model predictive ability. We focused on monkeypox occurrences in Central Africa for this test (West African sample sizes were not adequate to permit subsampling), separating them into 4 quadrants based on their locations above and below the median longitude and latitude on the overall data set from Central Africa (21.733°E and 0.387°S, respectively). Two quadrants (e.g., the northeast and southwest quadrants) were used to train ENMs, and occurrences in the other two quadrants (northwest and southeast) were used as an independent test of model predictability. The test was then repeated reversing training and testing quadrants. Binomial probabilities were used to assess the degree to which observed levels of agreement exceeded expectations under null models of no assumed association.

### Characterization and Comparison of Ecological Niches

In addition to the overall ENM, we developed ENMs for West and Central African occurrences separately to assess whether the two monkeypox clades occur in humans under similar ecological conditions. Sample sizes were divergent—146 from Central Africa *versus* 8 from West Africa (two adjacent localities in south-central Nigeria were excluded from this analysis, as viral specimens were unavailable for genetic analysis, and so could not be characterized as belonging to either West or Central African clades of monkeypox virus [15].

To permit visualization of ecological niches of various sets of occurrences analyzed, we related the geographic projection of the ENMs to the original environmental data layers to reconstruct ecological variation across the landscape and the conditions under which the species was predicted to be able to occur. To accomplish this, an intermediate table had to be constructed, which linked ENM predictions with environmental conditions pixel by pixel. This table was then exported in ASCII format and imported into programs for graphing [25].

## Results

**Figure 1:**
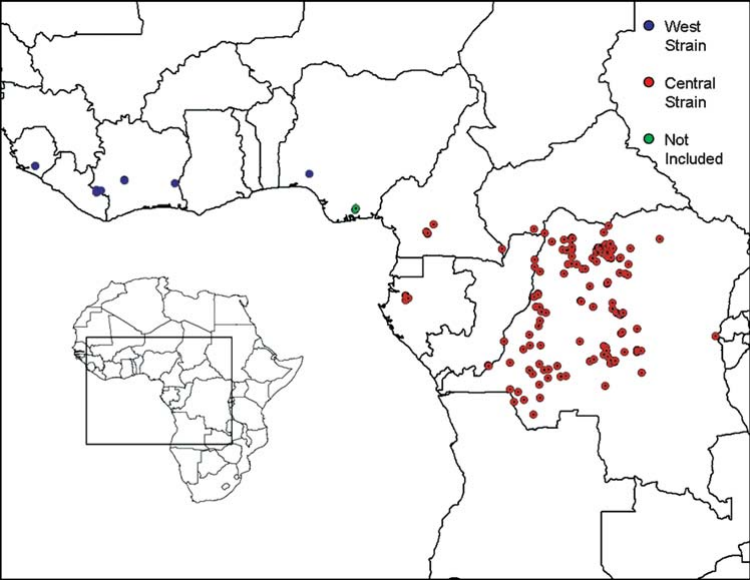
Occurrence locations of human monkeypox. Circles show partitioning of the 156 point occurrences into Central African (red), West Africa (blue), and unclassified (green) monkeypox genotypes.

The human monkeypox occurrence data set consisted of 156 unique localities, including 8 from West Africa, 146 from Central Africa (N = 146), and 2 from Nigeria that could not be assigned unambiguously to one group or the other for lack of viral specimens for molecular characterization [9] (**Figure 1**).

**Figure 2:**
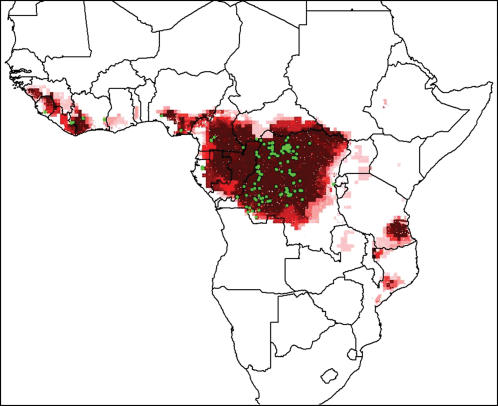
Overall predicted distribution of human monkeypox based on ecological niche modeling. Darker shades indicate areas with greater model agreement in prediction of suitability for monkeypox. Green points indicate input occurrences used in model development.

Using the full set of human monkeypox occurrences, the overall ENM (**Figure 2**) predicted potential distributional areas over most humid forest areas of Africa, including much of the DRC, Republic of Congo, Cameroon, and Gabon. A break in predicted favorable habitat occurs in western Cameroon and western Nigeria, leaving isolated patches of predicted potential distributional area in Ghana, Togo, Ivory Coast, Liberia, Sierra Leone, and Guinea. In East Africa, several isolated locations in Tanzania, Mozambique, and Madagascar are also predicted as potentially suitable.

The relative contribution of each environmental dimension to the overall predictive model of human monkeypox occurrence was assessed using the jackknife procedure (**Table 1**). All layers except ‘frost days’ (*p* = 0.11) were found to have statistically significant contributions to the model but exclusion of annual mean precipitation, flow direction, and land cover resulted in the greatest deviations, suggesting that these layers had substantial influence on the model. Overall, this analysis pointed strongly to annual mean precipitation as the key environmental dimension in model, as its exclusion resulted in a substantial drop in agreement with the model (0.63) and the largest deviation in mean pixel matches (−1.48).

**Table 1:**
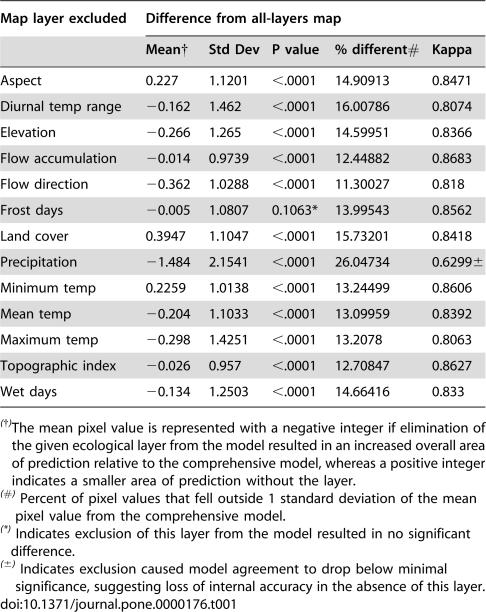
Summary of statistical analysis of ‘jackknife procedure’ used to determine environmental importance of ecological parameters (environmental layers).

Map layer excluded	Difference from all-layers map				
	Mean†	Std Dev	P value	% different#	Kappa
Aspect	0.227	1.1201	<.0001	14.90913	0.8471
Diurnal temp range	−0.162	1.462	<.0001	16.00786	0.8074
Elevation	−0.266	1.265	<.0001	14.59951	0.8366
Flow accumulation	−0.014	0.9739	<.0001	12.44882	0.8683
Flow direction	−0.362	1.0288	<.0001	11.30027	0.818
Frost days	−0.005	1.0807	0.1063*	13.99543	0.8562
Land cover	0.3947	1.1047	<.0001	15.73201	0.8418
Precipitation	−1.484	2.1541	<.0001	26.04734	0.6299±
Minimum temp	0.2259	1.0138	<.0001	13.24499	0.8606
Mean temp	−0.204	1.1033	<.0001	13.09959	0.8392
Maximum temp	−0.298	1.4251	<.0001	13.2078	0.8063
Topographic index	−0.026	0.957	<.0001	12.70847	0.8627
Wet days	−0.134	1.2503	<.0001	14.66416	0.833

(†) The mean pixel value is represented with a negative integer if elimination of the given ecological layer from the model resulted in an increased overall area of prediction relative to the comprehensive model, whereas a positive integer indicates a smaller area of prediction without the layer.

(#) Percent of pixel values that fell outside 1 standard deviation of the mean pixel value from the comprehensive model.

(*) Indicates exclusion of this layer from the model resulted in no significant difference.

(±) Indicates exclusion caused model agreement to drop below minimal significance, suggesting loss of internal accuracy in the absence of this layer.

**Figure 3:**
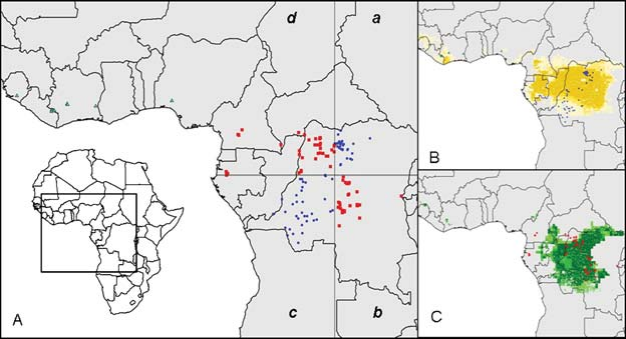
Summary of quadrant analyses of model robustness. (A) Central African human monkeypox occurrences divided into quadrants above and below the median longitude and latitude. (B) Modeled distribution created using only off-diagonal (*b/d*, red circles) input data. (C) Modeled distribution created using only on-diagonal (*a/c*, blue circles) input data. Darker shades indicate areas with greater model agreement in predicting area as suitable for monkeypox. Human monkeypox occurrence locations indicated by triangles were not used in model construction.

The quadrant tests allowed us to examine the generality and predictive ability of the ENMs developed in this study. We divided Central Africa into four quadrants (**Figure 3**), and generated ENMs based on two of them, to be tested via occurrences in the other two. In general, ENMs based on two of the four quadrants were able to predict most of the independent occurrence points—54 of 75 points for on-diagonal quadrants predicting off-diagonal quadrants, and 57 of 71 points for off-diagonal quadrants predicting on-diagonal quadrants; this degree of predictive ability was unexpectedly good, as compared with null hypotheses of no association (binomial probabilities, both *P*<0.001). These results suggest that the ENMs are robustly predicting the general picture of occurrences of human monkeypox cases, even into broad areas for which no input data are available.

**Figure 4:**
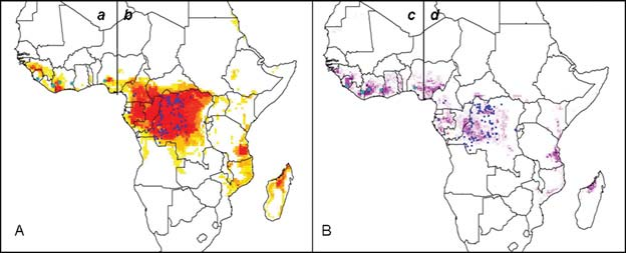
Ecological niche modeling results for West and Central African monkeypox. Eight West African and 146 Central African human monkeypox occurrences are indicated with green and blue circles, respectively. (A) Modeled distribution created using only Central African (blue) input data; (B) Modeled distribution created using only West African (green) input data. The vertical line (7.33°E longitude) denotes division between West and Central Africa. Nigerian occurrences were excluded from model development. Darker shades indicate areas with greater model agreement in prediction of potential suitability for monkeypox.

To assess whether West and Central African human monkeypox cases occupy similar or divergent ecological niches, ENMs were developed for each region individually, and projected to the other region (**Figure 4**). The Central African ENM predicted 6 of 8 West African points successfully, and the West African ENM predicted 45 of 146 Central African occurrences; both results were significantly higher levels of agreement between niche projections and independent test occurrence data than would be expected by chance (binomial tests, both *P*<0.001). The ENM generated based only on the 8 monkeypox occurrences from West Africa predicted a smaller overall area for monkeypox in Central Africa. Nonetheless, these results suggest that the ecological niche of human monkeypox cases is generally similar in West and Central Africa.

**Figure 5:**
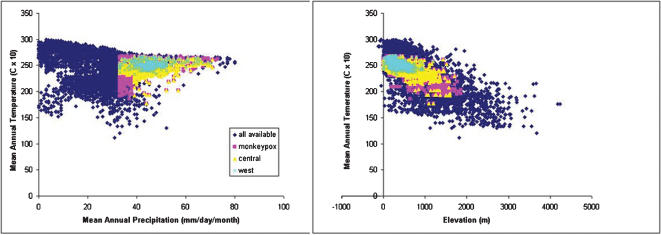
Visualizations of modeled monkeypox virus ecological niches in two-dimensional environmental spaces. Shown are all available habitat in the area of observation (dark blue diamonds); comprehensive monkeypox niche (pink); Central African niche (yellow), West African niche (light blue).

The general characteristics of observed human monkeypox occurrence locations in West and Central Africa are summarized in **Table 2**, where several differences are evident—e.g., elevation and maximum precipitation are lower in West African occurrences, and minimum temperature is higher. A final exploratory step in visualizing modeled African human monkeypox niches in environmental dimensions involved assessment of how predicted use relates to availability of conditions across landscapes in both West and Central Africa. **Figure 5** shows visualizations in 3 dimensions that illustrate broad trends. In general, West African monkeypox is shown to occupy a restricted subset of conditions relative to Central African monkeypox cases, but overall the two ENMs coincide closely. The sharp break observable in the figure for mean annual precipitation (**Figure 5**) indicates a concrete decision rule chosen by the GARP algorithm; this pattern is consistent with results of the jackknife analysis, in which removal of precipitation from ENM development resulted in large increases in area predicted habitable.

**Table 2:**
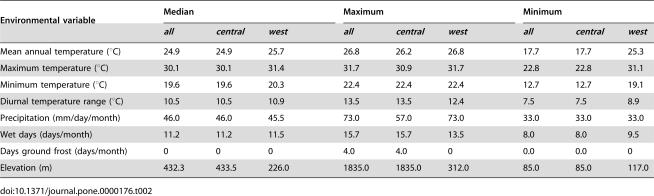
Values for climatic environmental variables at 156 geo-referenced points of human monkeypox occurrences.

Environmental variable	Median			Maximum			Minimum		
	*all*	*central*	*west*	*all*	*central*	*west*	*all*	*central*	*west*
Mean annual temperature (°C)	24.9	24.9	25.7	26.8	26.2	26.8	17.7	17.7	25.3
Maximum temperature (°C)	30.1	30.1	31.4	31.7	30.9	31.7	22.8	22.8	31.1
Minimum temperature (°C)	19.6	19.6	20.3	22.4	22.4	22.4	12.7	12.7	19.1
Diurnal temperature range (°C)	10.5	10.5	10.9	13.5	13.5	12.4	7.5	7.5	8.9
Precipitation (mm/day/month)	46.0	46.0	45.5	73.0	57.0	73.0	33.0	33.0	33.0
Wet days (days/month)	11.2	11.2	11.5	15.7	15.7	13.5	8.0	8.0	9.5
Days ground frost (days/month)	0	0	0	4.0	4.0	0	0.0	0.0	0
Elevation (m)	432.3	433.5	226.0	1835.0	1835.0	312.0	85.0	85.0	117.0

## Discussion

Our human monkeypox ENMs predict potential distributional areas for the disease throughout much of the Congo Basin, including parts of the DRC, Republic of Congo, Gabon, Central African Republic, Equatorial Guinea, and Cameroon, as well as small regions in West Africa in Nigeria, Ivory Coast, Sierra Leone, Liberia, Ghana, and Togo. This prediction generally coincides with the distribution of humid lowland evergreen tropical forest across Africa. Similar to parallel studies of Ebola virus distributions) [26], the model also identifies areas of potentially favorable habitat in East Africa–outside of regions of known human monkeypox disease occurrence–in Tanzania, Madagascar, and Mozambique. Interpretation of such disjunct potential distributional areas is complex—intervening areas that are apparently not favorable may limit dispersal of the host of monkeypox virus, and the virus may thus not occur in these areas [20]. The distributional area of monkeypox virus predicted by this model is based fundamentally on past identification and reporting of human cases. Any substantial under-reporting of (locally endemic) cases, particularly from geographic areas outside the current zone of prediction, would potentially alter our results. However, there is little evidence to suggest that endemic human monkeypox disease exists beyond the boundaries identified in this study.

The ENMs presented herein were generated using an evolutionary-computing approach designed to capture the broad ecological conditions associated with human monkeypox occurrence. The result is a predictive model rather than an observationally determined ecologic niche. Several empirical tests of internal predictive ability, however, indicate that the models are quite robust and general—that is, that they can anticipate the geographic distributions of independent data sets used in testing. A potential criticism of this approach for determining disease distribution is that little new information is generated, and that the analysis simply leads to “finding the forests,” in this case, the humid evergreen tropical forests of Africa. However, several lines of evidence contradict this notion—rather a bounded ecological space associated with presence of monkeypox virus has been identified *within* forested areas. This is suggested by the identification of mean annual precipitation rather than land cover as the key factor in the predicted monkeypox niche, as well as inclusion of effects of elevation and aspect as influential elements in the model. These restricted conditions likely point to a reservoir species, an intermediate host, or particulars of transmission that are highly dependent upon the same parameters.

To date, the reservoir species(s) of monkeypox virus remains undetermined. While previous studies have examined various species of African rodents and primates as possible reservoir species, imported rodents were implicated in the 2003 outbreak of monkeypox that occurred in the United States of America. Given our assumption that endemic human disease distribution in Africa is likely to coincide with the reservoir or intermediate host species distribution, the predicted map of human monkeypox presented here should assist in the determination of likely reservoir candidates [26], [27]. In addition, understanding the boundaries of probable endemic disease occurrence should help to guide public health investigators in to determining whether outbreaks of human monkeypox are likely due to an introduced or locally circulating source of virus.

Our ENMs may also provide insights into other aspects of human monkeypox disease outbreaks. Although current molecular genetic and epidemiologic evidence indicates the existence of biologically and genetically distinct monkeypox virus forms (14,15) in West and Central Africa, they are very similar ecologically. Subtle differences can be seen in ecological conditions associated with human monkeypox in the two regions—in general, human monkeypox in West Africa occurs in hotter, wetter conditions, and at lower elevations than in the Congo Basin—but these differences could simply reflect differences in habitat availability between the two regions or could result from the very small sample size of occurrences available to us from West Africa for model development [28].

In this study, ENM technology was used to predict the distribution of human monkeypox, and to identify ecological factors putatively associated with disease occurrence. The results of this study should impact our understanding of naturally-occurring human monkeypox and influence our expectations of where it may occur, and which species are likely to serve as transmission vehicles of the virus to humans. This study illustrates both the strengths and challenges of the ENM approach in understanding biological phenomena in remote and poorly studied regions. The small sample sizes of monkeypox case localities available—particularly for West Africa—may not reflect accurately the real prevalence of the disease. On the other hand, the end product of the ENM approach can serve as the foundation for additional hypothesis-driven ecological and epidemiologic research.
